# Competition for Hydrogen Prevents Coexistence of Human Gastrointestinal Hydrogenotrophs in Continuous Culture

**DOI:** 10.3389/fmicb.2020.01073

**Published:** 2020-05-29

**Authors:** Nick W. Smith, Paul R. Shorten, Eric Altermann, Nicole C. Roy, Warren C. McNabb

**Affiliations:** ^1^School of Food and Advanced Technology, Massey University, Palmerston North, New Zealand; ^2^Riddet Institute, Massey University, Palmerston North, New Zealand; ^3^AgResearch, Ruakura Research Centre, Hamilton, New Zealand; ^4^AgResearch, Grasslands Research Centre, Palmerston North, New Zealand; ^5^High-Value Nutrition National Science Challenge, Auckland, New Zealand; ^6^Liggins Institute, The University of Auckland, Auckland, New Zealand

**Keywords:** microbiome, hydrogen, mathematical modelling, methane, hydrogen sulphide, acetate, cross-feeding

## Abstract

Understanding the metabolic dynamics of the human gastrointestinal tract (GIT) microbiota is of growing importance as research continues to link the microbiome to host health status. Microbial strains that metabolize hydrogen have been associated with a variety of both positive and negative host nutritional and health outcomes, but limited data exists for their competition in the GIT. To enable greater insight into the behaviour of these microbes, a mathematical model was developed for the metabolism and growth of the three major hydrogenotrophic groups: sulphate-reducing bacteria (SRB), methanogens and reductive acetogens. In batch culture simulations with abundant sulphate and hydrogen, the SRB outcompeted the methanogen for hydrogen due to having a half-saturation constant 10^6^ times lower than that of the methanogen. The acetogen, with a high model threshold for hydrogen uptake of around 70 mM, was the least competitive. Under high lactate and zero sulphate conditions, hydrogen exchange between the SRB and the methanogen was the dominant interaction. The methanogen grew at 70% the rate of the SRB, with negligible acetogen growth. In continuous culture simulations, both the SRB and the methanogen were washed out at dilution rates above 0.15 h^−1^ regardless of substrate availability, whereas the acetogen could survive under abundant hydrogen conditions. Specific combinations of conditions were required for survival of more than one hydrogenotroph in continuous culture, and survival of all three was not possible. The stringency of these requirements and the inability of the model to simulate survival of all three hydrogenotrophs in continuous culture demonstrates that factors outside of those modelled are vital to allow hydrogenotroph coexistence in the GIT.

## Introduction

The human gastrointestinal tract (GIT) is home to a vast number of microbes that survive via metabolism of dietary and endogenous substrates, or via cross-feeding on molecules released by other members of the microbiota. Study of this metabolic network is challenged by the vast number of different strains and interactions present in the GIT microbiota of a single individual (Qin et al., 2010), as well as the inter-individual differences in microbiota profile and variation in this profile over time (Healey et al., 2017). However, the increasing number of links between diet, the microbiota and host health motivate greater understanding of the microbial community (Zmora et al., 2019).

For research purposes, the microbiota is often divided into functional groups based on metabolic substrates and products common to the group [for example, see Kalyuzhnyi and Fedorovich (1998), Motelica-Wagenaar et al. (2014), and Kettle et al. (2015)]. The metabolic activity of saccharolytic functional groups results in the release of hydrogen (Carbonero et al., 2012). The accumulation of hydrogen in the GIT environment reduces the efficiency of carbohydrate breakdown via inhibition of coenzyme reoxidation (Thauer et al., 1977; Wolin and Miller, 1983). Hydrogen is removed from this environment via host absorption and excretion, but also via hydrogenotrophic microbes. There are three major functional groups that metabolize hydrogen: the sulphate-reducing bacteria (SRB), the methanogens and the reductive acetogens. Each of these hydrogenotrophic functional groups or their metabolic products have been linked to nutritional and health impacts upon the host: hydrogen sulphide, produced by the SRB, has been investigated for its genotoxic effect on the GIT epithelium (Attene-Ramos et al., 2010); methane, produced by the methanogens, has been associated with constipation (Ghoshal et al., 2016); and acetate, produced by the acetogens, is readily absorbed by the host for use as an energy source (Morrison and Preston, 2016), but can also be cross-fed upon by other members of the microbiota (Falony et al., 2006). Numerous other links between hydrogenotrophs and the host have also been researched, including roles in irritable bowel syndrome, inflammatory bowel disease, colorectal cancer and possible impacts on obesity (for reviews, see Carbonero et al., 2012 and Smith et al., 2019b).

To investigate the competition for hydrogen between the three hydrogenotrophic functional groups, a mathematical model for their growth and metabolism in batch or continuous culture was developed. There exist models for each of these functional groups in monoculture and for certain co-culture combinations. A thermodynamics-based monoculture model for SRB growth and metabolism has been published (Noguera et al., 1998) and there exist numerous models for microbial methanogenesis both in monoculture and in co-culture with a SRB (see Junicke et al., 2016; Lynch et al., 2019, and Muñoz-Tamayo et al., 2019 for recent examples). Tamayo et al. (2008) and D'Hoe et al. (2018) have produced models for reductive acetogens in monoculture and co-culture, respectively. However, as yet there exists no model examining the interactions of all three hydrogenotrophs and we are not aware of any tri-culture data for this combination. Thus, the modelling presented here provides an unprecedented theoretical insight into the dynamics of these interactions. This model includes the hydrogenotrophic metabolic pathways of each group, as well as lactate oxidation by the SRB, which can be a source of hydrogen. Much previous research has demonstrated a hierarchy in hydrogen uptake efficiency between these groups, with SRB having the greatest affinity for hydrogen and the acetogens the least (for a review, see Smith et al., 2019b), but there is currently no experimental data for the direct competition between the three groups for this substrate. The hypothesis here was that the model would reveal what conditions were necessary for hydrogenotroph coexistence, as well as the conditions that would favour one group over the others.

## Materials and Methods

### Mathematical Model

The tri-culture model is the result of the additive combination of monoculture models for each of the three hydrogenotrophic functional groups. Both the SRB and acetogen components of the model are based on previously published model structures for *Desulfovibrio vulgaris* and *Blautia hydrogenotrophica*, respectively (Smith et al., 2019a, 2020), with minor notation alterations and the removal of mass transfer from the SRB model. Mass transfer is not included in the tri-culture model as the emphasis was on the ultimate outcome of cultures under various conditions, rather than the dynamics of the culture over time. The model also assumes that the culture media is homogeneously mixed, with no spatial component considered. The model structure is described below, with notation summarized in Table 1.

**Table 1:** Mathematical notation used in the model.

**Variables**	**Notation**	**Value (if a parameter)**	**Units**
Lactate concentration	*L*		mM
Sulphate concentration	*S*		mM
Hydrogen concentration	*H*		mM
Acetate concentration	*A*		mM
H_2_S concentration	*P*		mM
Methane concentration	*M*		mM
Concentration of SRB cells	*X*_*SRB*_		g L^−1^
Concentration of methanogen cells	*X*_*MET*_		g L^−1^
Concentration of acetogen cells	*X*_*ACE*_		g L^−1^
**RATE TERMS**			
Lactate metabolism by SRB	L		mM h^−1^
Sulphate metabolism by SRB	S		mM h^−1^
Hydrogen metabolism by the methanogen	M		mM h^−1^
Hydrogen metabolism by the acetogen	A		mM h^−1^
Dilution rate	*D*		h^−1^
Inflow rate of metabolite variable *i*	*I*_*i*_		mM h^−1^ (since *i* denotes a metabolite)
**SRB PARAMETERS**			
Maximum growth rate for lactate	μ_*max, L*_	0.116	h^−1^
Maximum growth rate for sulphate	μ_*max, S*_	0.03	h^−1^
Growth yield during growth on lactate	*Y*_*SRB, L*_	0.00565	g L^−1^ mM^−1^
Growth yield during growth on sulphate	*Y*_*SRB, S*_	0.00445	g L^−1^ mM^−1^
Hydrogen inhibition parameter	*H*_*max*_	0.0216	mM
Half-saturation constant for lactate uptake	*K*_*L*_	4.5	mM
Half-saturation constant for sulphate uptake	*K*_*S*_	0.05	mM
Half-saturation constant for hydrogen uptake	*K*_*SRB, H*_	1.69 × 10^−5^	mM
Moles of hydrogen produced per mole lactate used	*b*_*LH*_	2.5	–
Moles of hydrogen used per mole H_2_S produced	*b*_*HP*_	5	–
Moles of acetate produced per mole lactate used	*b*_*LA*_	1	–
Moles of H_2_S produced per mole sulphate used	*b*_*SP*_	1	–
**METHANOGEN PARAMETERS**			
Maximum growth rate for hydrogen	μ_*max, H*_	0.1042	h^−1^
Growth yield during growth on hydrogen	*Y*_*MET*_	0.0016	g L^−1^ mM^−1^
Half-saturation constant for hydrogen uptake	*K*_*MET, H*_	10.63	mM
Moles of methane produced per mole hydrogen used	*b*_*HM*_	0.0126	^−^
**ACETOGEN PARAMETERS**			
Threshold parameter	*p*_1_	0.015	mM^−1^
Threshold parameter	*p*_2_	336	mM
First order kinetics rate parameter	η	0.0054	h^−1^ mM^−1^
Growth yield during growth on hydrogen	Y_*ACE*_	0.0017	g L^−1^ mM^−1^
Moles of acetate produced per mole hydrogen used	*b*_*HA*_	0.25	–

The following metabolic pathways are assumed for each hydrogenotroph:

Lactate metabolism by the SRB (Noguera et al., 1998): CH_3_CHOHCOO^−^ (Lactate) + 2 H_2_O → CH_3_COO^−^ (Acetate) + 2 H_2_ + H^+^ + ${\text{HCO}}_{3}^{-}$ (Bicarbonate)

Sulphate metabolism by the SRB (Noguera et al., 1998): ${\text{SO}}_{4}^{2-}$ (Sulphate) + 5 H_2_ → H_2_S (Hydrogen sulphide) + 4 H_2_O

Hydrogen metabolism by the methanogen (Samuel et al., 2007): 4 H_2_ + CO_2_ → CH_4_ (Methane) + 2 H_2_O

Hydrogen metabolism by the acetogen (Schiel-Bengelsdorf and Dürre, 2012): 4 H_2_ + 2 CO_2_ → CH_3_COO^−^ (Acetate) + H^+^ + 2 H_2_O

Note that the tri-culture model does not consider the concentrations of H_2_O, CO_2_, bicarbonate or H^+^; these molecules are assumed abundant where required as substrates. In the case of the SRB, it is considered that 2.5 H_2_ are produced, rather than 2H_2_ + H^+^ (Smith et al., 2019a).

Let L and S represent the rate of lactate and sulphate metabolism by the SRB, respectively:

(1) $$\begin{array}{l} L=\frac{u_{\max,L}}{Y_{SRB,L}}\frac{L}{L+K_{L}}\left(1-\frac{H}{H_{max}}\right)X_{SRB} \end{array}$$

(2) $$\begin{array}{l} S=\frac{u_{max,S}}{Y_{SRB,S}}\frac{S}{S+K_{S}}\frac{H}{H+K_{SRB,H}}X_{SRB} \end{array}$$

These model equations are based on Monod kinetics (Monod, 1949), with the addition of a hydrogen inhibition term in the lactate metabolism equation (see Table 1 for definition of variables and parameters).

Next, let M and A be the rate of hydrogen metabolism by the methanogen and the acetogen respectively:

(3) $$\begin{array}{l} M=\frac{u_{max,H}}{Y_{MET}}\frac{H}{H+K_{MET,H}}X_{MET} \end{array}$$

(4) $$\begin{array}{l} A=\frac{\eta }{Y_{ACE}}\frac{H}{1+\exp\left(p_{1}\left(p_{2}-H\right)\right)}X_{ACE} \end{array}$$

The rate of hydrogen metabolism by the acetogen is not based on Monod kinetics, but rather on first order kinetics with the incorporation of a threshold modelling term, adapted from Ribes et al. (2004). This term ensures a rapid decrease in hydrogen uptake at concentrations below *p*_2_, with negligible uptake for concentrations below 70 mM, a previously derived threshold value for hydrogen uptake by the acetogen *B. hydrogenotrophica* (Leclerc et al., 1997; Smith et al., 2020). Using these rate terms, the full model is defined as the following system of ordinary differential equations (ODEs):

(5) $$\begin{array}{l} \frac{dL}{dt}=-L \end{array}$$

(6) $$\begin{array}{l} \frac{dS}{dt}=-S \end{array}$$

(7) $$\begin{array}{l} \frac{dH}{dt}=b_{LH}L-b_{HP}S-M-A \end{array}$$

(8) $$\begin{array}{l} \frac{dA}{dt}=b_{LA}L+b_{HA}A \end{array}$$

(9) $$\begin{array}{l} \frac{dP}{dt}=b_{SP}S \end{array}$$

(10) $$\begin{array}{l} \frac{dM}{dt}=b_{HM}M \end{array}$$

(11) $$\begin{array}{l} \frac{dX_{SRB}}{dt}=Y_{SRB,L}L+Y_{SRB,S}\;S \end{array}$$

(12) $$\begin{array}{l} \frac{dX_{MET}}{dt}=Y_{MET}M \end{array}$$

(13) $$\begin{array}{l} \frac{dX_{ACE}}{dt}=Y_{ACE}A \end{array}$$

Note that when the model is applied to continuous culture conditions, the following terms are appended to each ODE:

$$\begin{array}{l} -Di+I_{i} \end{array}$$

Where *D* is the dilution rate, *i* denotes the state variable of the ODE to which the term is appended and *I*_*i*_ is the inflow rate of *i*. In the cases considered here, *I*_*i*_ is set to zero for all microbial state variables; only metabolite inflows are permitted to be non-zero.

### Parametrisation of the Methanogen Model

Since the model for the methanogen is not based on a previously published model, a Monod model was fitted to monoculture experimental data. Data was obtained from Khelaifia et al. (2013) using graphical input and image capturing software in MATLAB (The MathWorks; www.mathworks.com). Cell concentration in mg ml^−1^ was determined using the optical density (OD) conversion of: mg ml^−1^ = 0.462 · OD (Richards et al., 2016). Although this was originally calculated for *Methanococcus maripaludis* at a different wavelength, there was difficulty in finding reliable conversion factors for *Methanobrevibacter smithii*. Khelaifia and Drancourt (2012) previously published a methanogen conversion factor of 0.4 · OD = 4.42^12^ cells/ml, which was calculated as approximately mg ml^−1^ = 0.236 · OD. However, the strain measured by Khelaifia and Drancourt (2012) was unclear and the calculation required an approximation on the weight of individual cells, therefore the more reliable conversion factor of Richards et al. (2016) was used. The two conversion factors were similar, implying that the true value is close to this range.

**Figure 1:**
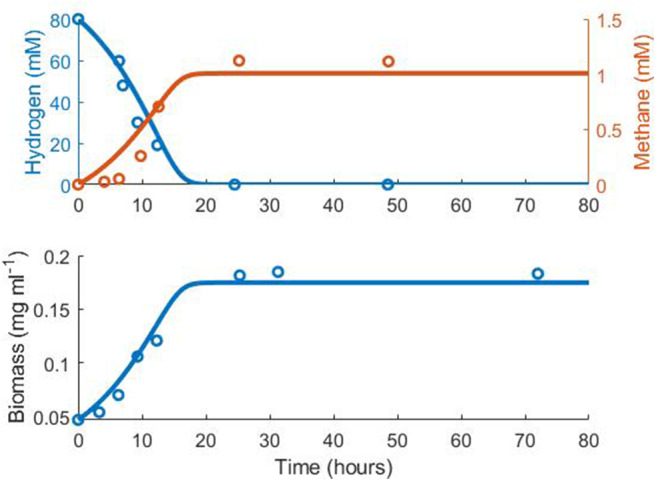
Fit of the methanogen model to experimental data from Khelaifia et al. (2013). Hydrogen *R*^2^ = 0.75, Methane *R*^2^ = 0.50, Biomass *R*^2^ = 0.85.

Notably, the expected stoichiometry of 1 mole methane produced per 4 moles hydrogen consumed was not observed in the experimental monoculture data (Figure 1, Table 1). It is unclear why the yield of methane was reduced in this experiment. However, the kinetic parameters obtained from fitting were comparable with estimates in the literature (Muñoz-Tamayo et al., 2019). As the stoichiometric yield of methane has no influence on the interactions between the three hydrogenotrophs in the model, the fitted value was used in later modelling.

### Numerical Simulations

For numerical analysis, the mathematical model was solved using the ode15s solver in MATLAB (The MathWorks; www.mathworks.com).

## Results

### Analysis of the Model Under Batch Culture Conditions

A comparison of the half-saturation and threshold parameter values for the three hydrogenotrophs shows the hierarchy in affinities for this substrate: the model estimates for the hydrogen half-saturation constants of the SRB and the methanogen are 1.69 × 10^−5^ mM and 10.63 mM, respectively (Table 1), while the threshold for hydrogen uptake for the acetogen is estimated at around 70 mM (Leclerc et al., 1997; Smith et al., 2020). As has been established by previous research, SRB generally have a greater affinity for hydrogen than the methanogens, which in turn have a greater affinity than the reductive acetogens for this substrate (for reviews, see Carbonero et al., 2012 and Smith et al., 2019b). For the acetogen in this model, growth limitation due to reduced substrate availability increases rapidly once the hydrogen concentration is below 336 mM. Using the parameter values in Table 1, the growth rate of the acetogen at this hydrogen concentration is 0.0069 h^−1^, which is small compared to the corresponding methanogen growth rate of 0.09 h^−1^ at this hydrogen concentration.

**Figure 2:**
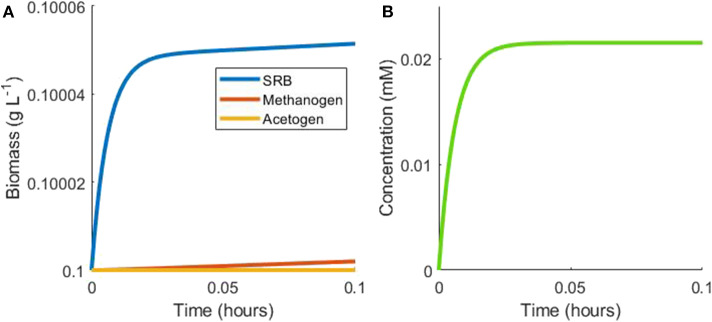
Example simulation of early stages of batch tri-culture with lactate the sole added substrate. **(A)** Shows the change in microbial biomass over time and **(B)** shows the change in hydrogen concentration over time. The SRB rapidly oxidizes lactate to acetate and hydrogen, resulting in SRB growth and hydrogen accumulation. Once hydrogen accumulates to a level approaching the inhibitory concentration for SRB growth, its concentration remains in a pseudo-steady state with a balance between hydrogen production by the SRB and consumption by the methanogen.

The complexity of the model equations, defined in the Materials and Methods section, precludes an analytical solution, but steady state analysis may still be performed. The first case considered is the batch culturing of all three hydrogenotrophs where lactate is the sole available substrate in the model. Under batch conditions with initially abundant lactate, the SRB will metabolize lactate to acetate and hydrogen rapidly, until the inhibitory hydrogen concentration is approached and growth is slowed (Figure 2). Growth would be halted completely under these conditions, but for the consumption of hydrogen by the other two hydrogenotrophs.

The acetogen's threshold for hydrogen uptake is greater than the inhibitory hydrogen concentration for the SRB: at the inhibitory hydrogen concentration for SRB, the growth rate of the acetogen, $A=4.41\;\times 10^{-4}X_{ACE}$, is very small, resulting in minimal acetogen growth under these conditions. The SRB inhibitory hydrogen concentration is also almost three orders of magnitude smaller than the half-saturation constant for hydrogen uptake by the methanogen, thus limiting methanogen growth, with methanogen growth rate of $M=0.13\;X_{MET}$.

A pseudo-steady state is then reached for the hydrogen concentration at a level approaching the inhibitory concentration, which is maintained by low-rate hydrogen consumption by the methanogen and hydrogen production by the SRB. Assuming this steady state and thereby nullifying $\frac{dH}{dt}$, the growth rate of the methanogen is proportional to that of the SRB at this steady state:

$$\begin{array}{l} 0=b_{LH}L-M\;\Rightarrow \;\frac{dX_{MET}}{dt}=\frac{b_{LH}Y_{MET}}{Y_{SRB,L}}\frac{dX_{SRB}}{dt}\approx 0.7\frac{dX_{SRB}}{dt} \end{array}$$

**Figure 3:**
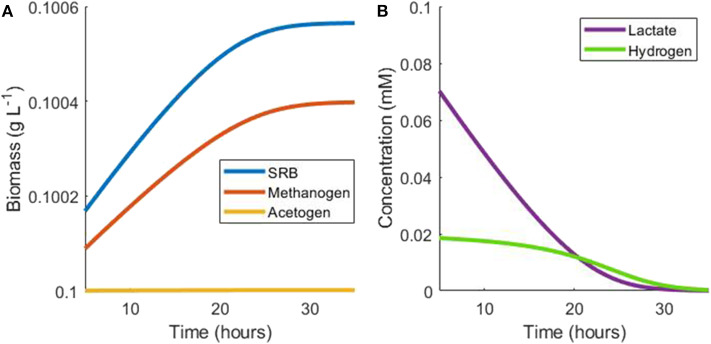
Example simulation of late stages of batch tri-culture with lactate the sole added substrate. **(A)** Shows the change in microbial biomass over time and **(B)** shows the change in hydrogen and lactate concentrations over time. As lactate is depleted, hydrogen production ceases and ultimately both lactate and hydrogen are depleted by the SRB and the methanogen, respectively.

Using the monoculture parameter values in Table 1 and assuming negligible growth by the acetogen, the growth rate of the methanogen is around 70% of the SRB growth rate in this situation. The population continues in this hydrogen steady state until lactate becomes depleted, at which point SRB growth is halted. As a result, hydrogen production ceases and hydrogen is then depleted by the methanogen, ultimately halting its growth (Figure 3).

If sulphate is also present in the medium in excess, hydrogen does not reach the inhibitory concentration due to its further use in the reduction of sulphate (data not shown). Again, negligible acetogen growth is possible at such a low hydrogen concentration. In numerical simulations (data not shown), the methanogens achieved only an incremental increase in biomass from hydrogen metabolism in this scenario, due to the hydrogen concentration remaining much lower than the methanogen half-saturation constant.

If hydrogen is the sole model substrate available, then the SRB will not grow, due to the absence of sulphate. Both the methanogen and the acetogen grow under these conditions, but the methanogen will show greater growth at low hydrogen concentrations due to its greater affinity for this substrate. At high hydrogen concentrations, the model predicts greater growth by the acetogen, since its growth rate increases linearly with the hydrogen concentration, whereas the methanogen's growth rate is limited to μ_max,*H*_. However, the acetogen model was parameterized at hydrogen concentrations below 200 mM, therefore likely extrapolates poorly to concentrations above this range (Smith et al., 2020). For higher hydrogen concentrations, it is likely that a Monod formulation would be more appropriate in capturing the acetogen dynamics. However, such high concentrations are not expected in the GIT (Carbonero et al., 2012; Wolf et al., 2016), hence the use of the threshold model.

Under our assumptions on the simple tri-culture batch scenario, the acetogen achieves a growth rate of at least *G* g L^−1^ h^−1^ if the hydrogen and acetogen cell concentrations satisfy

(14) $$\begin{array}{l} X_{ACE}>G\frac{1+\exp\left(p_{1}\left(p_{2}-H\right)\right)}{\eta H} \end{array}$$

**Figure 4:**
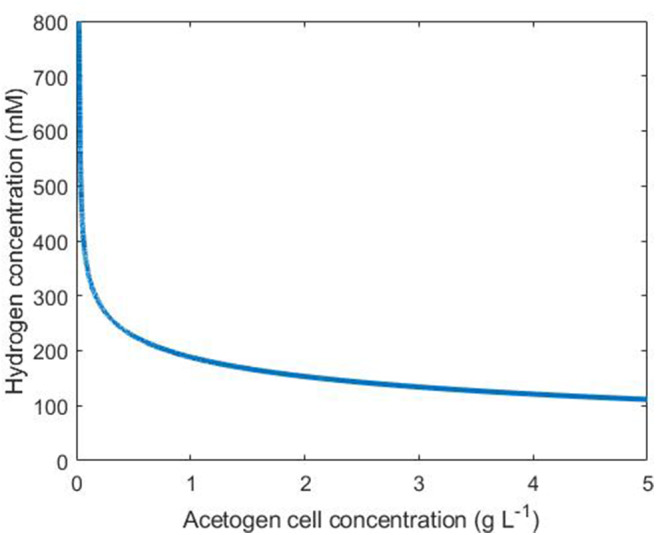
Conditions necessary for an acetogen growth rate above 0.1 g L^−1^ h^−1^. The line indicates the threshold for this growth rate: any combination of hydrogen and cell concentrations falling to the upper/right side of this line results in a growth rate above 0.1.

An interpretation of Equation 14 is shown in Figure 4, where points above and to the right of the line indicate conditions resulting in an acetogen growth rate of at least *G* = 0.1.

It is important to note that in the batch tri-culture environment, the hydrogen concentration will rapidly decrease due to metabolism by the methanogen and the SRB (if sulphate is available). Therefore, the acetogen will only display growth in the early stages of culture when hydrogen is abundant.

Table 2 summarizes the outcomes of various initial culture conditions on batch culture growth of the three hydrogenotrophs.

**Table 2:** Outcomes of batch culture under various initial conditions.

	**Hydrogenotrophs in culture at initial time**						
		**SRB + Methanogen + Acetogen**	**SRB + EITHER Methanogen OR Acetogen**	**Methanogen + Acetogen**	**SRB only**	**Methanogen only**	**Acetogen only**
Substrates available at initial time	Lactate + Sulphate + Hydrogen	Growth of SRB, minimal growth of methanogen and negligible growth of acetogen due to out-competition for hydrogen	Growth of SRB, minimal growth of other due to out-competition for hydrogen				
	Lactate + Sulphate	Growth of SRB, minimal growth of methanogen and negligible growth of acetogen due to out-competition for hydrogen	Growth of SRB, minimal growth of other due to out-competition for hydrogen				
	Lactate + Hydrogen	Growth of SRB when *H*<*H*_max_, minimal growth of methanogen and negligible growth of acetogen due to out-competition for hydrogen until *H*>*H*_max_, at which point SRB growth halted, methanogen and acetogen growth	Growth of SRB when *H*<*H*_max_, growth of other		Growth when *H*<*H*_max_		
	Sulphate + Hydrogen	Growth of SRB, minimal growth of others due to out-competition for hydrogen	Growth of SRB, minimal growth of other due to out-competition for hydrogen				
	Lactate only	Slow growth of all as SRB dependent on hydrogen removal by others. More growth by methanogen than acetogen due to low hydrogen concentration	Slow growth of both as SRB dependent on hydrogen removal by other		Growth until *H* reaches *H*_max_		
	Sulphate only						
	Hydrogen only	No growth of SRB, growth of methanogen, minimal growth of acetogen due to out-competition for hydrogen	No growth of SRB, growth of other				

*Green represents growth of all organisms, red represents no growth of any organisms, tan represents more complex dynamics*.

### Analysis of the Model Under Continuous Culture Conditions

Once dilution is introduced to the model, competition between the hydrogenotrophs for hydrogen becomes important in determining their survival. However, it is initially of importance to investigate what dilution rates allow for survival of each microbe individually. For a microbe to have the potential to survive continuous culture, its maximum growth rate must be greater than the dilution rate. The maximum growth rates of the SRB and the methanogen are 0.146 h^−1^ and 0.1042 h^−1^, respectively (Table 1), so they cannot survive in continuous culture with a greater dilution rate than these values. The maximum growth rate of the reductive acetogen is not as straightforward since under the current model structure it is determined in part by the hydrogen concentration. However, an acetogen growth rate of 0.1 h^−1^ would be achieved at a hydrogen concentration of 188 mM, therefore hydrogen concentrations exceeding this value would be required for the acetogen to survive a dilution rate (*D*) above 0.1 h^−1^.

When growing solely by sulphate reduction in the absence of lactate, the maximum growth rate of the SRB is 0.03 h^−1^. Washout of this bacterium therefore occurs at dilution rates exceeding this value, regardless of the abundance of hydrogen and sulphate.

The first scenario considered is competition for hydrogen between the methanogen and the acetogen. This is analyzed via their respective non-trivial steady states. Assuming abundant hydrogen and a steady state cell concentration for the methanogen, it can be seen that:

$$\begin{array}{l} D=\mu_{\max,H}\frac{H}{H+K_{MET,H}}\Rightarrow H=\frac{K_{MET,H}D}{\mu_{\max,H}-D} \end{array}$$

Similarly, assuming steady state for the acetogen gives:

(15) $$\begin{array}{l} D=\frac{\eta H}{1+\exp\left(p_{1}\left(p_{2}-H\right)\right)} \end{array}$$

**Figure 5:**
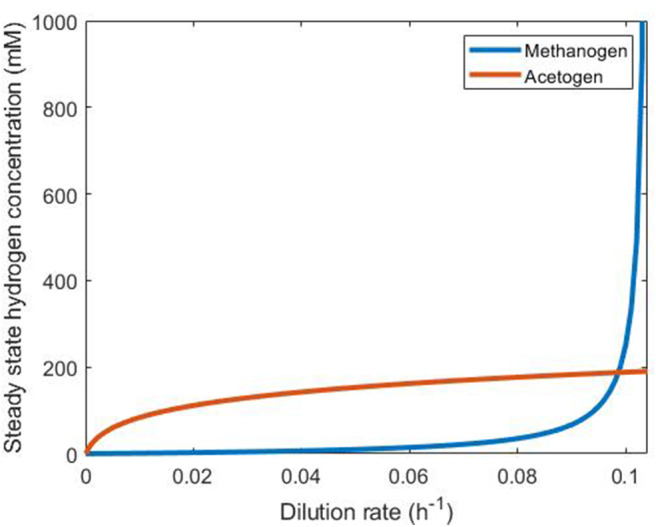
Comparison of steady state hydrogen concentrations achieved by the methanogen and the reductive acetogen. The hydrogenotroph with the lower of the two steady state hydrogen concentrations at a given dilution rate will outcompete the other for this substrate and survive while the other is washed out. Beyond D = 0.1042, the methanogen growth rate cannot be greater than the dilution rate, so it is washed out irrespective of the presence of the acetogen.

Plotting the dilution rate against the steady state hydrogen concentration (Figure 5) allows determination of the persistence of each of these two hydrogenotrophs under abundant hydrogen conditions in continuous culture. The microbe that achieves the lower steady state hydrogen concentration at a given dilution rate will outcompete the other by reducing the concentration of this substrate below its competitor's steady state value, leading to washout of the competitor. It can be seen in Figure 5 that the methanogen is therefore the survivor at lower dilution rates, and the acetogen at greater dilution rates. The only dilution rate at which the two may coexist is when their steady state hydrogen concentrations intersect, at a dilution rate of ~0.09861 h^−1^. At this dilution rate, the methanogen and the acetogen can coexist, given abundant hydrogen availability.

The more diverse metabolic capabilities of the SRB mean that conditions for SRB survival are more complex. If no lactate is available to the SRB, then survival requires that the dilution rate *D* ≤ *u*_max,*s*_ = 0.03, the maximum growth rate of the SRB in the absence of lactate. Moreover, a non-negative SRB growth rate, $\frac{dX_{SRB}}{dt}\geq 0$, requires that

(16) $$\begin{array}{l} D\leq \mu_{\max,S}\frac{S}{S+K_{S}}\frac{H}{H+K_{SRB,H}} \end{array}$$

**Figure 6:**
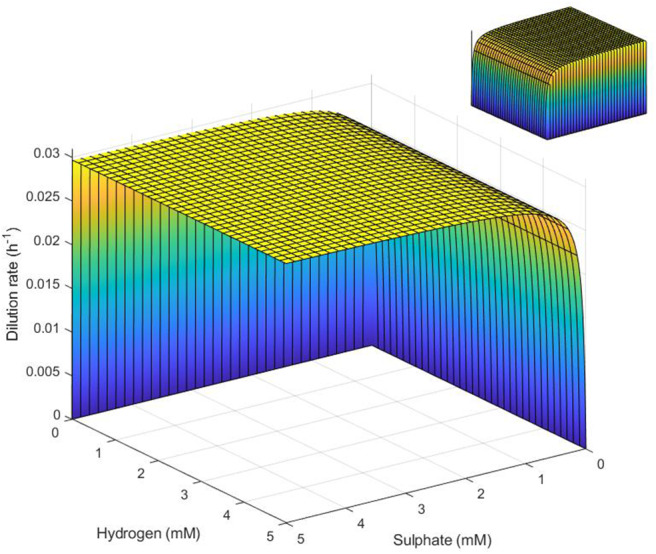
Surface representation of conditions necessary for SRB survival in the absence of lactate. SRB survival requires that conditions represent a point below the surface. Note the inverted sulfate and hydrogen axes. Inset: reverse view.

The surface plot in Figure 6 demonstrates these conditions on the three key variables.

If lactate is available in the medium, but sulphate is not, survival requires that *D* ≤ *u*_max,*L*_ = 0.116, the maximum growth rate of the SRB on lactate. Secondly, SRB growth is also impossible for hydrogen concentrations above *H*_max_. Thirdly, there is also the requirement that

(17) $$\begin{array}{l} D\leq u_{\max,L}\frac{L}{L+K_{L}}\left(1-\frac{H}{H_{\max}}\right)\leq u_{\max,L}\left(1-\frac{H}{H_{\max}}\right) \end{array}$$

(18) $$\begin{array}{l} \Rightarrow H\leq H_{\max}\left(1-\frac{D}{u_{\max,L}}\right) \end{array}$$

**Figure 7:**
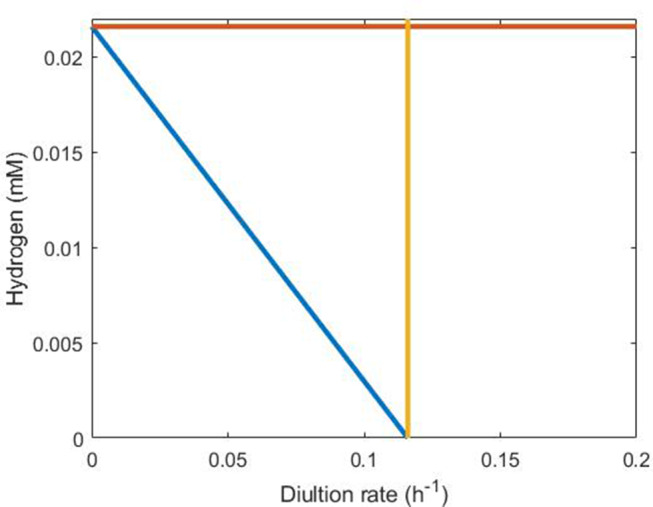
Representation of conditions necessary for SRB survival in the absence of sulfate. The orange line indicates *H* = *H*_max_, the yellow line indicates *D* = 0.116 and the blue line indicates the inequality derived in the text (Equation 18) between dilution rate and hydrogen concentration. SRB survival requires that steady state conditions lie below and to the left of all three lines, making the blue line clearly the strongest requirement.

Figure 7 shows these conditions graphically using the parameter values in Table 1. Clearly, the third condition encapsulates the first two and is the most stringent under these parameter values.

We may also extract further, more complex conditions for SRB growth when lactate is the sole added substrate. Taking the steady state for the hydrogen differential equation under abundant lactate, no hydrogen inflow and with only the SRB present allows derivation of the steady state hydrogen concentration:

$$\begin{array}{l} \;0=b_{LH}\frac{\mu_{\max,L}}{Y_{SRB,L}}X_{SRB}\left(1-\frac{H_{SS}}{H_{\max}}\right)-DH_{SS}\;\\ \Rightarrow H_{SS}=b_{LH}\frac{\mu_{\max,L}}{Y_{SRB,L}}X_{SRB}{\left(D+b_{LH}\frac{\mu_{\max,L}}{H_{\max}Y_{SRB,L}}X_{SRB}\right)}^{-1} \end{array}$$

Substituting this value into the equation for the SRB growth rate under abundant lactate conditions and simplifying gives the SRB growth rate at steady state as:

$$\begin{array}{l} \mu_{\max,L}\left(1-\frac{H_{SS}}{H_{\max}}\right)=\frac{DH_{\max}\mu_{\max,L}Y_{SRB,L}}{\left(DH_{\max}Y_{SRB,L}+b_{LH}\mu_{\max,L}X_{SRB}\right)} \end{array}$$

Since this growth rate must be greater than or equal to the dilution rate to prevent washout, we may derive the following condition for SRB survival:

(19) $$\begin{array}{l} D\leq \mu_{\max,L}\left(1-\frac{b_{LH}X_{SRB}}{H_{\max}Y_{SRB,L}}\right)\approx 0.116-2376X_{SRB} \end{array}$$

Therefore, survival of the SRB requires that the cell concentration is < ~ 4.88 × 10^−5^ g L^−1^; any greater concentration would result in too rapid an accumulation of hydrogen and subsequent SRB inhibition. This constitutes a very small window for successful SRB continuous culture when lactate is the sole added substrate.

Moving on to the survival of co-cultures including the SRB, next analyzed is the coexistence of the SRB and the methanogen when no lactate is available. In this case, the steady state hydrogen concentrations for each in monoculture are

(20) $$\begin{array}{l} H_{SS,SRB}=\frac{K_{SRB,H}}{\frac{\mu_{\max,S}S}{D\left(K_{S}+S\right)}-1} \end{array}$$

for the SRB and

(21) $$\begin{array}{l} H_{SS,MET}=\frac{K_{MET,H}D}{\mu_{\max,H}-D} \end{array}$$

for the methanogen. Whichever of these values is smaller under a given set of conditions will indicate the hydrogenotroph that will survive, the other being outcompeted. Similar to the case for the methanogen and the acetogen, coexistence is possible where these two steady states coincide. When sulphate is assumed abundant, this occurs at *D* = 0.03, which is intuitive since this is the value of μ_max,*S*_. If sulphate is not abundant, then setting the two steady state equations above equal to one another gives

(22) $$\begin{array}{l} D\approx \frac{0.03S}{0.05+S} \end{array}$$

Thus, as the steady state sulphate concentration (*S*) decreases, so too must *D* to allow for continued coexistence of both the SRB and the methanogen. For dilution rates lower than this value, the SRB will out-compete the methanogen for hydrogen, with the converse true for dilution rates above this value.

Following the same argument for the SRB and the acetogen, once again survival of both is possible under abundant sulphate at *D* = 0.03, with acetogens out-competed below this value and SRB out-competed above this value. An analytical solution requires the use of the Lambert *W* function for the case when sulphate is limiting due to the complexity of the acetogen growth rate equation, thus is not useful for this analytical analysis.

Next, in the case where lactate is available, but sulphate is not, clearly a steady state hydrogen concentration approaching the inhibitory concentration will be maintained if both the SRB and the methanogen are present, analogously to the batch culture case. Assuming a steady state hydrogen concentration of *H*_max_, we have the growth rate for the methanogen of

(23) $$\begin{array}{l} M=\frac{\mu_{\max,H}}{Y_{MET}}\frac{H}{K_{H}+H}X_{MET}=\frac{\mu_{\max,H}}{Y_{MET}}\frac{H_{\max}}{K_{H}+H_{\max}}X_{MET}\\ \;\approx 0.132X_{MET} \end{array}$$

Thus, the dilution rate must be less than this growth rate for the methanogen to survive. Under these conditions, SRB growth will be limited by the hydrogen consumption rate of the methanogen. If the methanogen is growing at the upper limit rate of 0.132*X*_*MET*_, then hydrogen is removed from the system at the rate:

$$\begin{array}{l} 0.132X_{MET}+DH_{\max} \end{array}$$

This is therefore the rate at which the SRB produces hydrogen to maintain the steady state. Thus:

(24) $$\begin{array}{l} b_{LH}\;L=0.132X_{MET}+DH_{\max} \end{array}$$

and since the rate of growth of the SRB is $Y_{SRB,L}L$, we have the SRB growth rate of:

(25) $$\begin{array}{l} Y_{SRB,L}L=\frac{Y_{SRB,L}}{b_{LH}}\left(0.132X_{MET}+DH_{\max}\right)\approx 2.98\times 10^{-4}X_{MET}\\ \;+4.88\times 10^{-5}D \end{array}$$

Since the dilution rate must be lower than the growth rate of both the SRB and the methanogen for coexistence to be sustained, we see that coexistence of the SRB and the methanogen in the presence of excess lactate is possible only when the dilution rate is very low and the methanogen cell concentration sufficiently high.

Following the same argument, we may also derive co-culture survival conditions for the SRB and the acetogen. In this case, the growth rate for the acetogen at the hydrogen inhibition concentration is $4.41\times 10^{-4}X_{ACE}$ (g L^−1^ h^−1^) and the corresponding SRB growth rate is $\sim 4.88\times 10^{-4}X_{ACE}+4.88\times 10^{-5}D$ (g L^−1^ h^−1^).

Table 3 outlines the outcomes of all possible culture combinations under excess substrate inflow based on the conclusions of the analytical derivations described previously. For the cases where multiple substrates are present, but at limiting concentrations, we turn to a computational analysis of outcomes.

**Table 3:** Outcomes of continuous culture under various conditions.

		**Hydrogenotrophs in culture at initial time**						
		**SRB + Methanogen + Acetogen**	**SRB + Methanogen**	**SRB + Acetogen**	**Methanogen + Acetogen**	**SRB only**	**Methanogen only**	**Acetogen only**
Substrates supplied in abundance to culture	Lactate + Sulphate + Hydrogen	Analogous to Sulphate + Hydrogen case as high hydrogen concentration prevents lactate metabolism by SRB	Analogous to Sulphate + Hydrogen case as high hydrogen concentration prevents lactate metabolism by SRB	Analogous to Sulphate + Hydrogen case as high hydrogen concentration prevents lactate metabolism by SRB	Coexistence at *D* = 0.09861, acetogen out-competes methanogen above this and methanogen out-competes acetogen below this	Survival if *D* ≤ *u*_max,*S*_ as lactate metabolism inhibited by high hydrogen concentration	Survival if *D* ≤ *u*_max,*M*_	
	Lactate + Sulphate	SRB survival if *D* ≤ *u*_max,*L*_+*u*_max,*S*_. Others washed out due to out-competition for hydrogen or no hydrogen production if SRB washed out	SRB survival if *D* ≤ *u*_max,*L*_+*u*_max,*S*_. Methanogen washout due to out-competition for hydrogen or no hydrogen production if SRB washed out	SRB survival if *D* ≤ *u*_max,*L*_+*u*_max,*S*_. Acetogen washout due to either out-competition for hydrogen or no hydrogen production if SRB washed out		Survival if *D* ≤ *u*_max,*L*_+*u*_max,*S*_		
	Lactate + Hydrogen	SRB washout as high hydrogen concentration prevents lactate metabolism. Methanogen and acetogen coexistence at *D* = 0.09861, acetogen out-competes methanogen above this and methanogen out-competes acetogen below this	Methanogen growth. SRB washout due to hydrogen concentrations above the inhibitory concentration	Acetogen growth. SRB washout due to hydrogen concentrations above the inhibitory concentration	Coexistence at *D* = 0.09861, acetogen out-competes methanogen above this and methanogen out-competes acetogen below this	Washout as lactate metabolism inhibited by high hydrogen concentration	Survival if *D* ≤ *u*_max,*M*_	
	Sulphate + Hydrogen	Coexistence of SRB and methanogen at *D* = 0.03, acetogen out-competed for hydrogen. SRB only below this value. Methanogen and acetogen coexistence at *D* = 0.09861. Methanogen only between these bounds, acetogen only above the upper bound	Coexistence only possible at *D* = 0.03. Methanogens out-competed for hydrogen at lower dilution rates, SRB washed out at higher dilution rates	Coexistence only possible at *D* = 0.03. Acetogens out-competed for hydrogen at lower dilution rates, SRB washed out at higher dilution rates	Coexistence at *D* = 0.09861, acetogen out-competes methanogen above this and methanogen out-competes acetogen below this	Survival if *D* ≤ *u*_max,*S*_	Survival if *D* ≤ *u*_max,*M*_	
	Lactate only	Analogous to SRB + Methanogen case, since acetogen's hydrogen threshold for meaningful growth is much higher than methanogen's. Either survival of SRB and methanogen only, or all are washed out.	Both survive if *D*/*X*_*MET*_ sufficiently small. Otherwise, SRB alone may survive if at steady state *D*, *X*_*SRB*_ and *H* satisfy boundary conditions, else both will be washed out	Both survive if *D*/*X*_*ACE*_ sufficiently small. Otherwise, SRB alone may survive if at steady state *D*, *X*_*SRB*_ and *H* satisfy boundary conditions, else both will be washed out		Survival if at steady state *D*, *X*_*SRB*_ and *H* satisfy boundary conditions		
	Sulphate only							
	Hydrogen only	SRB washout, Methanogen and acetogen coexistence at *D* = 0.09861. Methanogen only below this value, acetogen only above	SRB washout, methanogen survival if *D* ≤ *u*_max,*M*_	SRB washout, acetogen growth	Coexistence at *D* = 0.09861, acetogen out-competes methanogen above this and methanogen out-competes acetogen below this		Survival if *D* ≤ *u*_max,*M*_	

*Green represents growth of all organisms, red represents no growth of any organisms, tan represents more complex dynamics, with details given*.

### Numerical Analysis of the Model Under Continuous Culture Conditions

In order to investigate survival of the hydrogenotrophs under mixed culture conditions, substrate inflow and dilution rate ranges were examined. These ranges were chosen as they provide coverage of conditions that allow for growth of all three hydrogenotrophs individually and allow for examination of transitions in culture dominance between the microbes. Moreover, since the motivation for this study is the behavior of hydrogenotrophs in the human GIT, substrate concentrations comparable to concentrations expected in this environment were deemed appropriate. The substrate ranges investigated were: lactate 0–25 mM h^−1^ (Vernia et al., 1988; Macfarlane and Macfarlane, 2012; Pham et al., 2017); sulphate 0–10 mM h^−1^ (Florin et al., 1991; Lewis and Cochrane, 2007); and hydrogen 0–50 mM h^−1^ (Carbonero et al., 2012; Wolf et al., 2016). The range of dilution rates was 0.01–0.15 h^−1^, as only acetogen survival is possible above this range under current model assumptions. The substrate inflow ranges were split into 1 mM h^−1^ intervals, and the dilution rate range was split into 0.01 h^−1^ intervals. The mathematical model was then run for all 218,790 possible combinations of these inflow and dilution rates to establish whether each hydrogenotroph survived the culture conditions. Initial cell concentration for each hydrogenotroph was set at 1 g L^−1^, and the initial concentration of each of the three substrates was set at the maximum value of its range. Each microbe was determined to have survived if, after 1,000 h of simulated continuous culture, its cell concentration was >1 × 10^−4^ g L^−1^ and its population was not decreasing. The population was determined to be still decreasing if, over the final 20 h of simulation, the cell concentration decreased by more than 1 × 10^−9^ g L^−1^. If the microbe failed to satisfy these survival conditions, it was deemed extinct from the culture.

Of the 218,790 simulated metabolite inflow and dilution rate combinations, under no conditions were all three hydrogenotrophs coexisting after 1,000 h, as expected from the analytical investigation of the model. Similarly, under no conditions was coexistence of the acetogen with either of the other hydrogenotrophs possible. The only possible coexisting pair was the SRB and the methanogen. Each of the three hydrogenotrophs could outcompete the other two under certain conditions, and there were also many conditions under which all three were washed out.

**Figure 8:**
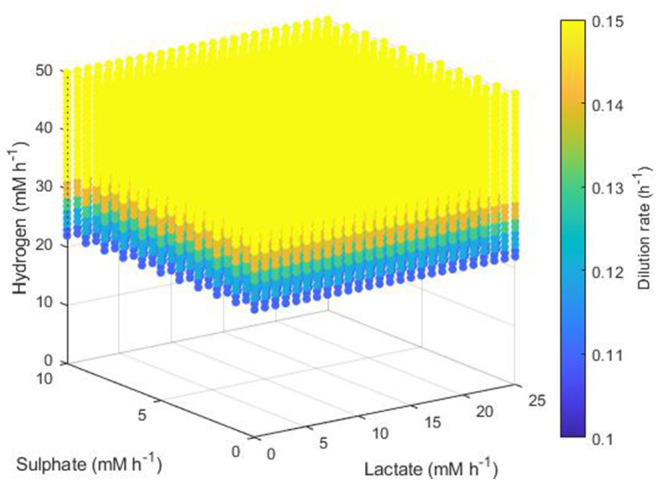
Maximum dilution rates (h^−1^) under which the outcome of the model was survival of the acetogen only. The axes indicate the inflow rate of each of the three substrates, and the colored points represent combinations of these inflows that resulted in survival of the acetogen only at the dilution rate shown on the color bar.

The simplest set of conditions to examine was those in which the acetogen outcompeted the other two hydrogenotrophs. Figure 8 shows the maximum dilution rates under which only the acetogen survived to 1,000 h. This survival was independent of the lactate and sulphate inflow, but required a hydrogen inflow of at least 22 mM h^−1^. As hydrogen inflow increased above this cut-off, the acetogen was able to outcompete the others and survive at greater dilution rates. Note that out-competition by the acetogen occurred only when the dilution rate was above 0.1 h^−1^; it was earlier stated that 0.146 h^−1^ and 0.1042 h^−1^ were the greatest dilution rates under which the SRB and methanogen could survive, respectively.

**Figure 9:**
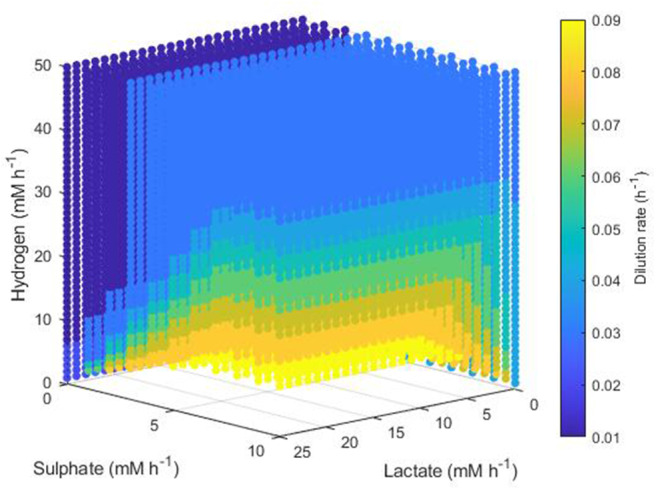
Minimum dilution rates under which the outcome of the model was survival of the methanogen only. The axes indicate the inflow rate of each of the three substrates, and the colored points represent combinations of these inflows that resulted in survival of the methanogen only at the dilution rate shown on the color bar. Note the inverted lactate and sulfate axes.

Conditions resulting in survival of only the methanogen took a more complex form. Figure 9 shows the minimum dilution rate under which only this microbe survives. At low lactate, low sulphate, high hydrogen inflows, the methanogen outcompetes the other hydrogenotrophs at most dilution rates. However, as lactate and sulphate inflows increase, only under higher dilution rates and hydrogen inflows can the methanogen outcompete the SRB; for combinations of very high lactate and sulphate inflows with very low hydrogen inflow, the methanogen is outcompeted by the SRB regardless of dilution rate. Conversely to Figure 8 for acetogen only survival, Figure 9 shows only dilution rates up to 0.09, as the methanogen is unable to avoid washout at dilution rates above 0.1042 h^−1^.

**Figure 10:**
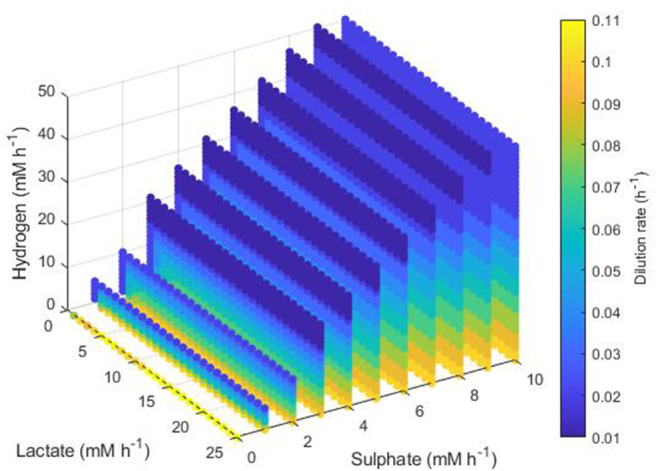
Maximum dilution rates under which the outcome of the model was survival of the SRB only. The axes indicate the inflow rate of each of the three substrates, and the colored points represent combinations of these inflows that resulted in survival of the SRB only at the dilution rate shown on the color bar. Note the inverted lactate axis.

Figure 10 shows the maximum dilution rate under which the SRB outcompetes the other hydrogenotrophs. When sulphate and lactate inflows were high and hydrogen inflow was low, the SRB was able to outcompete the other microbes at dilution rates up to 0.1 h^−1^. This result was unaffected by lactate inflow. However, as hydrogen inflow increases, inhibition of lactate utilization by the SRB increases also, thus the SRB were only able to outcompete at low dilution rates. The same was true for low sulphate inflows.

**Figure 11:**
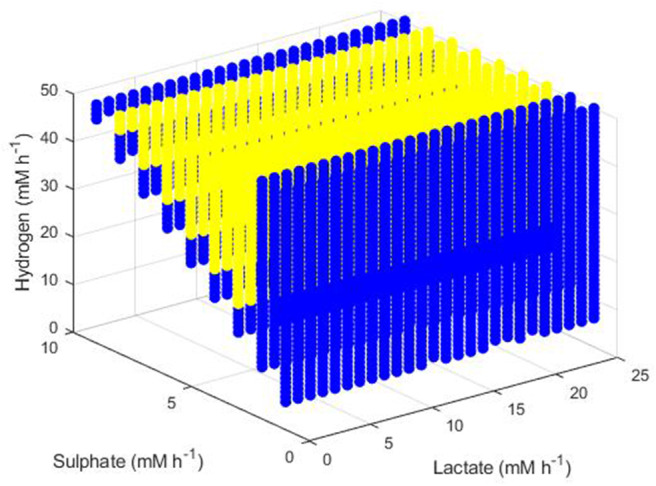
Dilution rates under which the outcome of the model was survival of the SRB and the methanogen. The axes indicate the inflow rate of each of the three substrates. Yellow points indicate conditions where coexistence was possible at both 0.01 and 0.02 h^−1^ dilution rates and blue points indicate conditions where coexistence was only possible at a dilution rate of 0.02 h^−1^.

The final set of conditions that did not result in extinction of all three hydrogenotrophs were those that enabled survival of both the SRB and the methanogen, as shown in Figure 11. The greatest dilution rate which resulted in this coexistence was 0.02 h^−1^, emphasizing the low growth rates achieved by both microbes at hydrogen concentrations approaching the inhibitory concentration, as shown earlier analytically. Coexistence was unaffected by lactate concentration, but did require a relationship between he sulphate and hydrogen inflows: high hydrogen inflows had to coincide with high sulphate inflows to maintain survival of both the SRB and the methanogen. Coexistence also required a minimum hydrogen inflow of 6 mM and was not possible at zero sulphate inflow. The blue region in Figure 11 indicates conditions under which a dilution rate of 0.01 h^−1^ did not result in coexistence, although this is likely a result of the model not reaching steady state after 1,000 h, as discussed below. It is anticipated that all colored points in Figure 11 represent conditions that would allow for survival of both the SRB and the methanogen at either 0.01 or 0.02 h^−1^ dilution rates.

The results shown in this numerical section, and in particular those shown in Figure 11, may not be reflective of steady state survival. Under our assumptions, these results merely indicate survival and very slow cell concentration change after 1,000 h of simulated culture. The low dilution rates considered may result in a longer time required for the model to reach steady state under certain conditions, so the above results are not necessarily steady state results in all cases. However, when the conditions resulting in SRB and methanogen coexistence were simulated for 2,000 h, the survival status was identical to the 1,000 h case, making steady state survival likely. Moreover, survival of a microbial population after 1,000 h likely translates to survival under practical, shorter timeframes.

## Discussion

A number of previous studies suggested that a degree of competitive exclusion exists between hydrogenotrophs when competing for hydrogen in the human GIT (Gibson et al., 1988a,b, 1990, 1993; Strocchi et al., 1994). However, more recent work has found that all three hydrogenotrophs are present in the GIT of all tested individuals (Nava et al., 2012). There exists a great body of research detailing the dominant hydrogenotroph in different human populations and in different locations in the GIT, as well as the influence of dietary changes on this dominance (for a review, see Smith et al., 2019b). The modelling work presented here shows that under conditions of abundant substrate in a homogeneously mixed environment, dominance of a culture by one species is the most common result, with extinction of the others. Moreover, under low-dilution, high-substrate conditions, the model results support the established hierarchy between SRB, methanogens and acetogens in terms of their growth rates and competitiveness.

The prediction of the model that coexistence of all three hydrogenotrophs in continuous culture is impossible under any of the substrate and dilution rate combinations considered is in contrast with their observation is natural habitats, such as the human GIT (Nava et al., 2012). However, the model assumption of a homogeneously mixed environment is likely partially responsible for this difference. In natural habitats, spatial separation, diffusion of metabolites, biofilm formation and many other heterogeneities will contribute both positively and negatively to the growth of each of the hydrogenotrophs. For example, being separated spatially from the methanogen would allow for better growth by the acetogen due to reduced local competition for hydrogen, whereas the same spatial separation from the SRB may be detrimental to the acetogen if it is reliant on cross-feeding for released hydrogen.

Also neglected in this model is the metabolism of substrates other than hydrogen, lactate and sulphate by the hydrogenotrophs. Several GIT SRB strains have been shown to metabolize other organic substrates (Willis et al., 1997), as have GIT acetogens (Bernalier et al., 1996). Formate is a molecule that has been shown to be metabolized by strains of all three hydrogenotrophs (Bernalier et al., 1996; Samuel et al., 2007; da Silva et al., 2013), and would be a valuable addition to the tri-culture model. However, the addition of this extra metabolite would make analytical solutions to the model more challenging to obtain, and numerical results more challenging to interpret.

It is not expected that the parameter values determined from monoculture experiments stated in Table 1 will be suitable for all hydrogenotroph strains in all environments, as gene expression changes between monoculture and co-culture have been shown in SRB and methanogen co-cultures previously (Walker et al., 2009; Meyer et al., 2013). The model results demonstrated that survival of more than one hydrogenotroph under continuous culture conditions was only possible under a small set of conditions, and we would not expect this qualitative result of the model to change given small variations to the parameter values. However, so long as the assumptions on the model system remain unchanged, the analytical results provided here will still apply. If the assumptions change or large variations to the parameter values are made, a revised analysis of the model would be required.

The applicability of the model to natural environments is also of interest. Coexistence of multiple hydrogenotrophs was shown to be possible only under a very limited set of conditions in the model. Small deviations due to external, non-modelled influences would be expected to occur in natural environments. Under the model conclusions, these small deviations would rapidly lead to extinction of all or all but one of the modelled organisms. However, the observed coexistence of hydrogenotrophs in natural environments indicates that other factors have an influence. In the human GIT, these other factors may be the use of alternative metabolites, spatial separation of hydrogenotrophs and interactions with the wider microbiota and host. Also not addressed by this model was the potential for metabolite influx to follow a periodic or inconsistent pattern, as would be expected in the GIT. The influence of all these factors on GIT survival of hydrogenotrophs should be included and investigated in future models.

Inclusion of the non-modelled influences mentioned above in future modelling efforts for hydrogenotroph metabolism in the GIT will be challenging, but the tools exist for such investigations. Several spatial models of microbial growth and metabolism have been developed, using both continuous (for example, see Alpkvist et al., 2006) and discrete spatial distribution (for example, see Bauer et al., 2017). There are also microbiome models that incorporate various host influences, such as pH buffering and water absorption (Cremer et al., 2017), the secreted mucus layer and fluid dynamics of the intestinal contents under peristaltic movement (Labarthe et al., 2019). Finally, the influence of interactions amongst the microbiota can be studied with existing microbial community models (for examples, see Motelica-Wagenaar et al., 2014; Kettle et al., 2017, and Sung et al., 2017). These models were more comprehensive and applicable to the GIT than that presented here, but hydrogenotrophic microbes have thus far been either absent or only a minor part of these large-scale models. Both the study of these microbes and the large-scale models themselves could benefit from the inclusion of hydrogenotrophs.

The complexity of the conditions for survival derived from the relatively simple model presented here, which features only three different organisms and six metabolites (three of which had no determining effect on the model outcomes), in a homogenously mixed medium with no gaseous phase, demonstrates the difficulty in extracting universal insight into large, complex populations from mathematical models. However, mathematical modelling has been used to successfully glean more information from experimental data for co- and tri-cultures of human GIT bacteria (Van Wey et al., 2014; Pinto et al., 2017; D'Hoe et al., 2018) and for cross-feeding cultures of SRB and methanogens in the past (Archer and Powell, 1985; Stolyar et al., 2007). These successes justify the further use of modelling to explore areas that cannot easily be studied experimentally. We are not aware of any research in which the three hydrogenotrophs modelled here have been tri-cultured together, thus the results of the model currently forms the best predictive capability available for what would be observed experimentally. Moreover, the simple nature of the constituent models will allow for straightforward inclusion of additional variables and conditions, such as formate metabolism. Although an increase in complexity will likely prevent analytical solutions to the model equations, model simulations can still provide useful insight into microbial community dynamics.

To conclude, the results of the mathematical model show that coexistence of all three hydrogenotrophs in the human GIT must be due to selective factors outside of the hydrogen, lactate and sulphate metabolism and varied dilution rates considered here, since it was impossible to obtain coexistence of the three in continuous culture under the model assumptions. The influence of these other selective factors needs to be examined in future models investigating hydrogenotroph dynamics in the GIT.

## Data Availability Statement

The raw data supporting the conclusions of this article will be made available by the authors, without undue reservation, to any qualified researcher.

## Author Contributions

All authors contributed to the conceptualization of the model. NS performed the modelling work and wrote the manuscript. All authors contributed to the revision of the manuscript.

## Conflict of Interest

The authors declare that the research was conducted in the absence of any commercial or financial relationships that could be construed as a potential conflict of interest.
